# Novel Gd Nanoparticles Enhance Vascular Contrast for High-Resolution Magnetic Resonance Imaging

**DOI:** 10.1371/journal.pone.0013082

**Published:** 2010-09-30

**Authors:** Tot Bui, Jeff Stevenson, John Hoekman, Shanrong Zhang, Kenneth Maravilla, Rodney J. Y. Ho

**Affiliations:** 1 Department of Pharmaceutics, University of Washington, Seattle, Washington, United States of America; 2 Department of Radiology, University of Washington, Seattle, Washington, United States of America; Karolinska Institutet, Sweden

## Abstract

**Background:**

Gadolinium (Gd), with its 7 unpaired electrons in 4f orbitals that provide a very large magnetic moment, is proven to be among the best agents for contrast enhanced MRI. Unfortunately, the most potent MR contrast agent based on Gd requires relatively high doses of Gd. The Gd-chelated to diethylene-triamine-penta-acetic acid (DTPA), or other derivatives (at 0.1 mmole/kg recommended dose), distribute broadly into tissues and clear through the kidney. These contrast agents carry the risk of Nephrogenic Systemic Fibrosis (NSF), particularly in kidney impaired subjects. Thus, Gd contrast agents that produce higher resolution images using a much lower Gd dose could address both imaging sensitivity and Gd safety.

**Methodology/Principal Findings:**

To determine whether a biocompatible lipid nanoparticle with surface bound Gd can improve MRI contrast sensitivity, we constructed Gd-lipid nanoparticles (Gd-LNP) containing lipid bound DTPA and Gd. The Gd-LNP were intravenously administered to rats and MR images collected. We found that Gd in Gd-LNP produced a greater than 33-fold higher longitudinal (T_1_) relaxivity, *r_1_*, constant than the current FDA approved Gd-chelated contrast agents. Intravenous administration of these Gd-LNP at only 3% of the recommended clinical Gd dose produced MRI signal-to-noise ratios of greater than 300 in all vasculatures. Unlike current Gd contrast agents, these Gd-LNP stably retained Gd in normal vasculature, and are eliminated predominately through the biliary, instead of the renal system. Gd-LNP did not appear to accumulate in the liver or kidney, and was eliminated completely within 24 hrs.

**Conclusions/Significance:**

The novel Gd-nanoparticles provide high quality contrast enhanced vascular MRI at 97% reduced dose of Gd and do not rely on renal clearance. This new agent is likely to be suitable for patients exhibiting varying degrees of renal impairment. The simple and adaptive nanoparticle design could accommodate ligand or receptor coating for drug delivery optimization and *in vivo* drug-target definition in system biology profiling, increasing the margin of safety in treatment of cancers and other diseases.

## Introduction

Magnetic resonance imaging (MRI) plays a pivotal role in non-invasive visualization and quantification of vascular and tissue pathology or physiologic processes. The use of paramagnetic contrast agents increases sensitivity and specificity of medical diagnoses based on MRI. The paramagnetic MR contrast agents modify the local magnetic environment due to interactions between unpaired electrons of contrast media, such as gadolinium (Gd^3+^), and the hydrogen nuclei of water in the blood, tissues and organs in the body. Among paramagnetic compounds, Gd is the primary agent used for MRI due to high relaxation efficiency and magnetic moments. As a free, soluble ionic metal ion, Gd^3+^ is highly toxic, probably due to its affinity for metalloproteins and calcium binding proteins. Therefore, Gd contrast agents are formulated as Gd bound chemical chelates that are water soluble to improve their clinical safety profile. Some of the clinically used Gd-chelates include DTPA (Magnevist), BOPTA (MultiHance), HP-DO3A (ProHance), DTPA-BMEA (Optimark), DTPA-BMA (Omniscan), DOTA (Dotarem), and DTPA-DPC (Vasovist or MS-325) (a partial list of chelates and their abbreviations are listed in reference 1).

It is clear that Gd-chelate-based MR contrast agents have allowed identification of pathologic tissue and physiological processes that are not detectable by other non-invasive imaging modalities. Due to wide-spread tissue and cell distribution, relatively high doses (0.03–0.1 mmole/kg) of Gd^3+^ in one of the above chelate forms are essential to produce sufficient MR image definition. However, available Gd-based contrast agents are eliminated predominantly through the kidney. Administration of Gd-chelates in patients with significantly reduced creatinine clearance or renal impairment is linked to nephrogenic systemic fibrosis (NSF) [1]. Data suggest that NSF is associated with increased cell and tissue exposure of Gd-chelates and Gd dissociation from chelates. Therefore, all FDA approved Gd contrast agents carry a ‘Black Box’ safety warning [2]. A Gd contrast agent that exhibits higher contrast potency at a much lower Gd dose that is effective for use in renal impaired subjects is urgently needed. Therefore, we explored the possibility of using a biocompatible lipid nanoparticle with surface bound Gd that can be retained in intact blood vasculature and can be eliminated by the biliary route to avoid reliance on renal function. This report describes our discovery of a high potency MR contrast agent that is built on lipid-nanoparticles with Gd bound to lipidic chelate, and its improved MRI contrast resolution and elimination profile. This approach could address both imaging sensitivity and safety of Gd use.

## Results

### Preparation and Characterization of Gd-lipid Nanoparticles

First, we determined whether sequestration of Gd^3+^ on the surface of lipid nanoparticles through DTPA chelate embedded in nanoparticle can enhance the MRI properties of Gd. To do so, we constructed lipid nanoparticles containing phospholipids that express Gd chelate or DTPA by incorporating DTPA-linked to phosphatidylethanolamine or DTPA-PE into the lipid core of the nanoparticles. The DTPA-PE was incorporated as 10% (m/m) of lipid nanoparticles constructed with disteroylphosphatidylcholine. Then Gd^3+^ (as Gd^3+^ in solution) was added to preformed lipid nanoparticles (d = 60–70 nm) expressing DTPA-PE (for binding to Gd^3+^ as Gd-DTPA-PE chelate). We found that at either 1∶1 or 1∶2 Gd-to-DTPA-PE molar ratio, all Gd^3+^ added in solution was bound to DTPA chelate molecules expressed on lipid nanoparticles without significantly affecting the diameter of the nanoparticles (Table 1). Complete association of Gd-to-DTPA-PE expressed on lipid nanoparticles was verified based on the ability of free Gd^3+^ to quench calcein fluorescence (λ_ex_ = 490 nm; λ_em_ = 520 nm). Results indicated that no free Gd^3+^ was available to quench calcein at both 1∶1 and 1∶2 Gd-to-DTPA-PE mole ratios. The complete chelating of Gd to lipid-nanoparticle expressing DTPA was also verified by gel-permeation chromatography (data not shown).

**Table 1:** Effects of varying the molecular weight of PEG expressed on Gd-lipid nanoparticles on Gd relaxivity constant and the particle diameter.

Gd-DTPA-nanoparticles or other formulations^a^	*r_1_* [mM^−1^_*_s^−1^]^b^	*r_2_* [mM^−1^_*_s^−1^]^b^	Diameter of particles (nm ± S.D.)^a^
**Nanoparticle with**			
mPEG5900	61.2	8.1	63±0.2
mPEG2000	134.8	12.7	70±0.6
mPEG750	21.8	3.9	64±0.2
Gd-DTPA-liposomes	13.6	5.3	88±0.4
Gd-DTPA-DPC Vasovist or MS 325	6.6 (28.0*)	—	Solution (*in rat serum)
Gd-DTPA Magnevist	3.8	2.4	Solution
Gd-DTPA-BMA Omniscan	4.0	3.1	Solution

a Gd-DTPA lipid nanoparticles containing 1,2-distearoyl-sn-glycero-3-phosphocholine (DSPC; 9 part), and 1,2- distearoyl-sn-Glycero-3-phophoethanolamine-N-DTPA (DSPE-DTPA; 1 part), and N-(Carbonyl-methoxypolethyleneglycol-polymer of listed molecular weight)-1,2-distearoyl-sn-glycero-3-phospoethanolamine (mPEG-DSPE, 1 part) in suspension were mixed with gadolinium (III) chloride hexahydrate (Gd^3+^, at 1∶1 DTPA:Gd mole ratio) for 20 minutes. For comparison, water soluble commercial agents such as Omniscan (Gd-DTPA-BMA) and Magnevist (GD-DTPA) were also included. The lipid particle diameter was expressed at mean ±a SD.

b The relaxation time T_1_ was measured using the standard spin-echo sequence on a 3T MR scanner. These concentration dependent T_1_ values were plotted versus Gd^3+^ concentration from which the rising curve was fitted by linear regression to estimate apparent molar relaxivity constant ***r_1_*** and ***r_2_***. The covariant of ***r1*** and ***r2*** data were 15% or lower.

Next, to increase the bound water on the lipid nanoparticle surface, we added 10 mole percentage of lipid conjugated to methyl-polyethylene-glycol or mPEG-PE to lipid nanoparticles. The molecular weight of mPEG-PE is varied to explore the role of surface bound water on Gd paramagnetic properties. Paramagnetic potency was evaluated as changes in T_1_ and T_2_, the longitudinal and transverse relaxation time constants for MRI. Compared to soluble Gd-DTPA or Gd-DTPA-BMA, we found that inclusion of mPEG on Gd-lipid nanoparticle surfaces significantly increased longitudinal T_1_ relaxivity, *r_1_* (Table 1). The Gd-lipid nanoparticles (Gd-LNP) containing mPEG_2000_-PE exhibited the highest increase in *r_1_*, recorded at 134.8 mM^−1^*s^−1^. This *r_1_* value is about 33-fold higher than that of Gd-DTPA-BMA (^r1^ = 4 mM^−1^*s^−1^), similar to the intrinsic value of Gd. Based on these data, we selected lipid nanoparticles containing mPEG_2000_-PE and Gd-DTPA-PE, referred to as Gd-LNP, for subsequent experiments.

**Figure 1:**
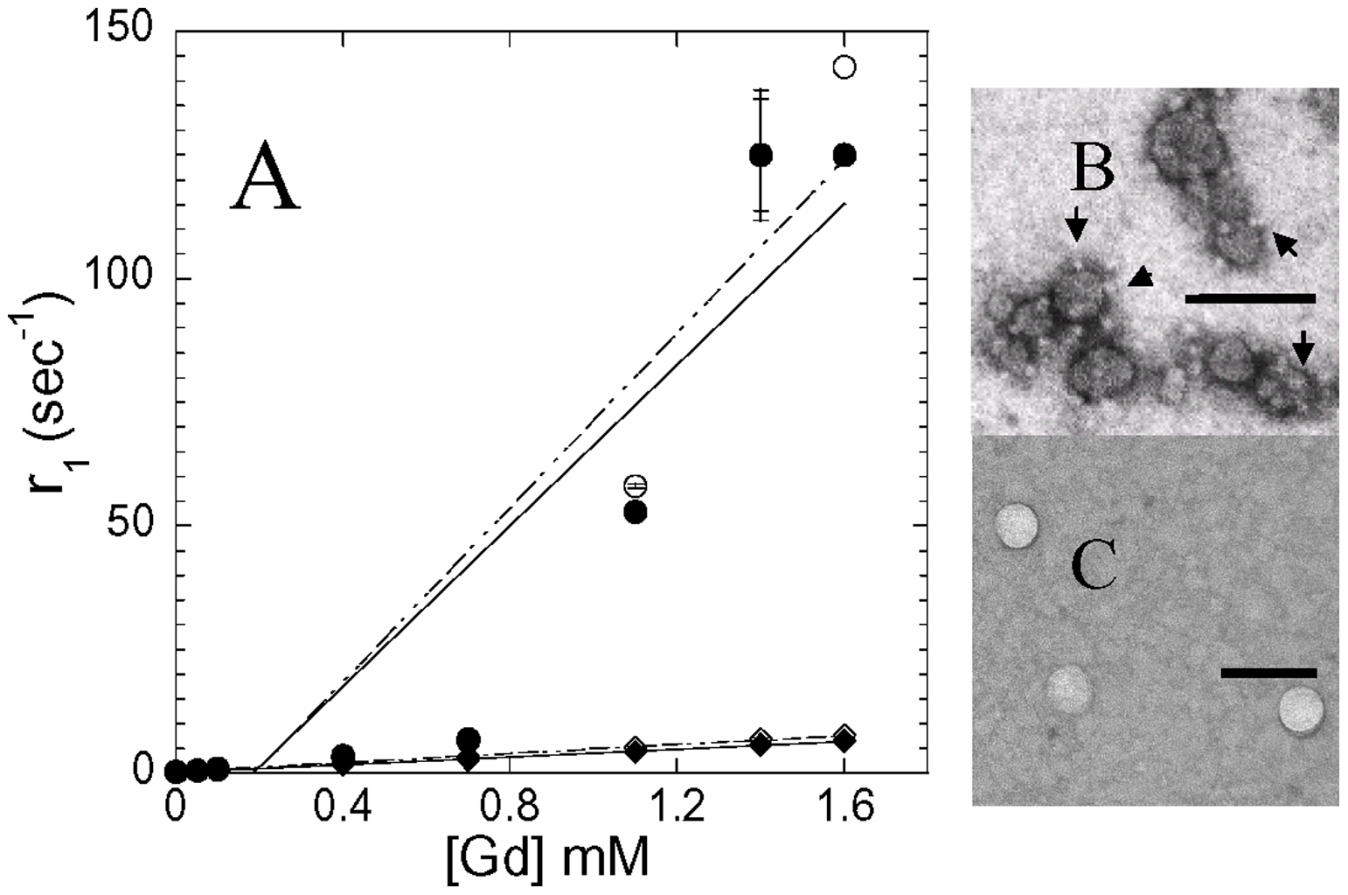
Effects of serum on Gd-concentration dependent change in longitudinal (T_1_) relaxation time and comparison of lipid-nanoparticle morphology between Gd-DTPA liposome and Gd-lipid nanoparticles. Panel A: Gd-lipid nanoparticles containing mPEG_2000_-PE (○,●) or Gd-DTPA liposomes without (mPEG) (◊,♦), were exposed to serum (●,♦) and relaxation time was measured with a 3T MRI instrument as described in Table 1. The data were fitted using linear regression. The electron micrographs represent morphology of Gd-lipid nanoparticles (panel B) and Gd-DTPA liposomes (panel C). Samples were negatively stained with 1% phosphotunstate. Please note the small electron exclusion bodies surrounding the Gd-lipid nanoparticles that were not detectable with Gd-DTPA liposomes. The bars in panels B and C represent 100 nm.

To evaluate effects of serum on Gd relaxivity, we first exposed the Gd-LNP to 50% rat serum for 1 hr. All Gd-LNP, regardless of the molecular weight of mPEG, as well as Gd-DTPA liposomes exhibit 63–70 nm in diameters (Table 1). Gd-nanoparticle diameters as determined by photon correlation spectroscopy, did not exhibit any significant change in particle size due to serum exposure. These particles were further evaluated for T_1_ relaxivity with a 3T MRI instrument. Analysis of Gd concentration-dependent relaxivity before and after serum exposure indicates that molar relaxivity of Gd-LNP was not influenced by serum (Figure 1). Morphology analysis of Gd-LNP by electron microscopy indicates distinct surface morphology compared to typical liposomes expressing DTPA-PE (Figure 1B vs. 1C). The Gd-LNP exhibited unique small electron exclusion bodies on lipid-nanoparticle surfaces (Figure 1B) that were not seen with typical lipid membrane vesicle or liposome preparations [3]. The morphology reported for Gd-DTPA liposomes (Figure 1C) are similar to those reported for PEG-expressed liposomes carrying either Gd or gold [3], [4]. This unique morphologic data is also consistent with the much higher relaxivity data collected in Table 1 for mPEG_2000_-PE expressed Gd-LNP (Table 1 and Figure 1).

### Effects of Gd Association to Gd-lipid Nanoparticles on MR Imaging in Rats

**Figure 2:**
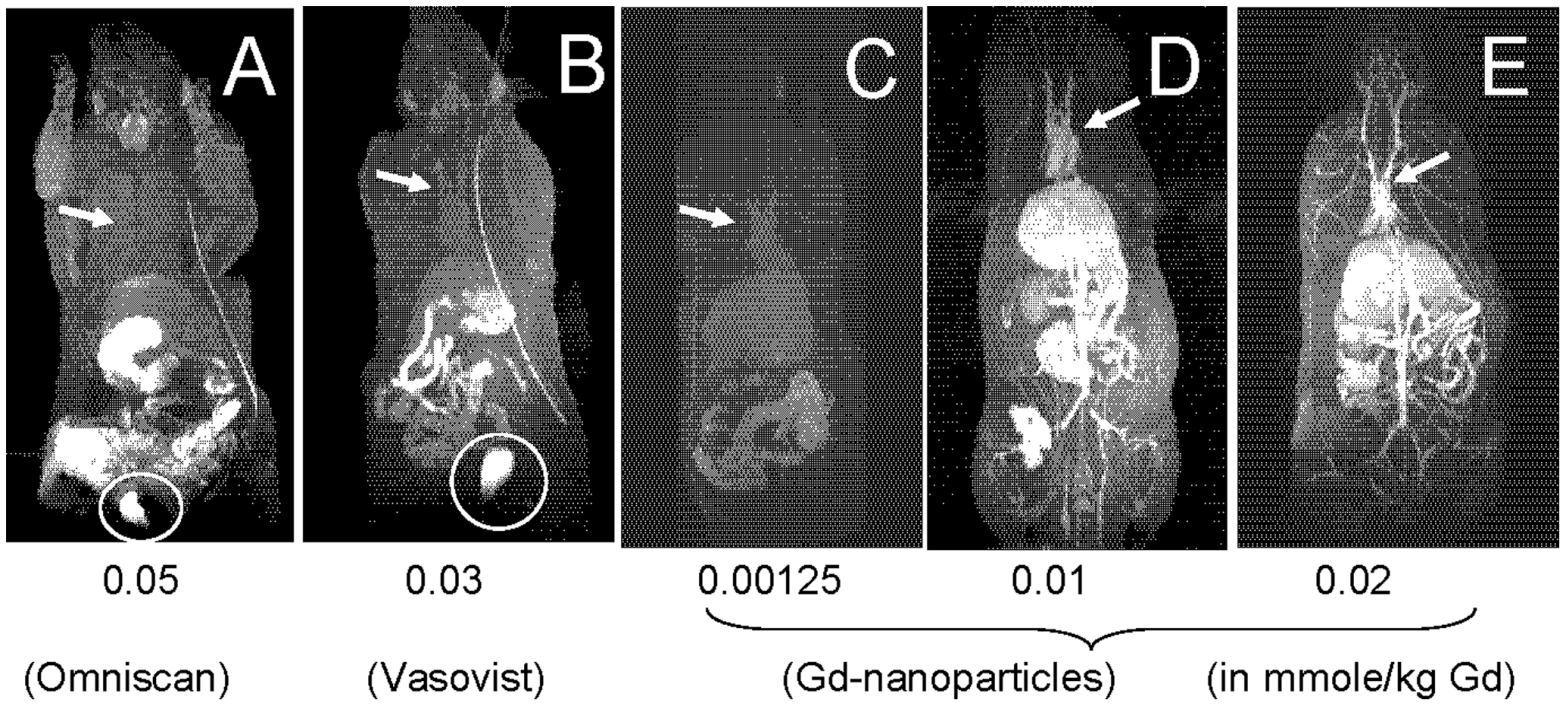
Comparison of whole body MR image obtained using a 3T MR instrument. The rats were intravenously given indicated doses of Gd, in Gd-DTPA-BMA (Omniscan, panel A), Gd-DTPA-DPC (Vasovist, panel B) or Gd-lipid nanoparticles (panels C-E). The mmole/kg dose of Gd were 0.05 for Omniscan (panel A), 0.03 for Vasovist (panel B), 0.00125, 0.01, 0.02 for Gd-lipid-nanoparticles (panels C, D & E, respectively). The DCE-MR images were collected at 15 min post Gd administration. The circles and arrows indicate Gd-dependent MR contrast in the bladder and the heart blood vessels. Also, the catheter line used for IV administration of Gd contrast is apparent as a high contrast line.

Subsequently, rats were administered with varying doses of the Gd-LNP intravenously and T_1_ weighted MR images were collected. At 15 minutes after 0.00125–0.02 mmole/kg Gd-LNP administration, a dose much lower than the two clinically approved agents Omniscan (Figure 2A) and Vasovist (Figure 2B), all blood vasculature including vessels in the liver, heart, and kidney were clearly apparent (Figure 2D–E). In contrast, at their respective clinical doses, relatively low resolution of heart, liver and some vasculature was noted with the FDA approved agents (Figure 2A–B vs. 2C–E). More importantly, the FDA approved Gd agents localized in the bladder, while no bladder accumulation of Gd in the rats administered Gd-LNP was detectable in MR images (Figure 2). In rats administered with 0.01 mmole/kg Gd-LNP (Figure 2D), the Gd dynamic-contrast enhanced (DCE) MRI analysis revealed a signal-to-noise (S/N) ratio greater than 300 in all vasculature (vasculature versus surrounding tissues). It provided much greater anatomical and vascular details at 0.00125 than that achieved with either 0.05 or 0.03 mmole/kg of Gd-DTPA-BMA (Omniscan) or Gd-DTPA-DPC (Vasovist or MS-325) (Figure 2). Thus, approximately 97% lower or only 3% of current clinical Gd dose in Gd-LNP is needed to produce equivalent or higher MRI contrast resolution.

We next evaluated the dose-response of Gd-LNP for MRI contrast properties. As shown in Figure 2 panels C-E, a lower Gd dose reduced contrast levels but provided better definition than that collected at higher doses of Omniscan or Vasovist. Even at 0.00125 mmole/kg Gd-LNP dose, clear definition of heart and liver as well as vasculature connecting tissues was apparent. The MR image collected at 0.00125 mmole/kg (or 1.25 µmole/kg) Gd-LNP dose (Figure 2C) is about equivalent to that collected with 0.03 mmole/kg Gd in the Vasovist formulation, and better than that collected with 0.05 mmole/kg Gd in the Omniscan formulation. Thus, compared to 0.05 mmole/kg Gd dose in Omniscan, only 2.5% [(0.00125/0.05) ×100%] of Gd in Gd-LNP is needed to produce higher quality MR images. The dose-response MR contrast data were replicated with another batch of Gd-LNP to verify these results. Collectively, based on the dose dependent data, contrast potency improvement is estimated to be at least 24-fold higher than Gd in the Vasovist formulation, which is considered the most sensitive vascular agent approved for clinical use [5].

### Kinetics and Clearance

**Figure 3:**
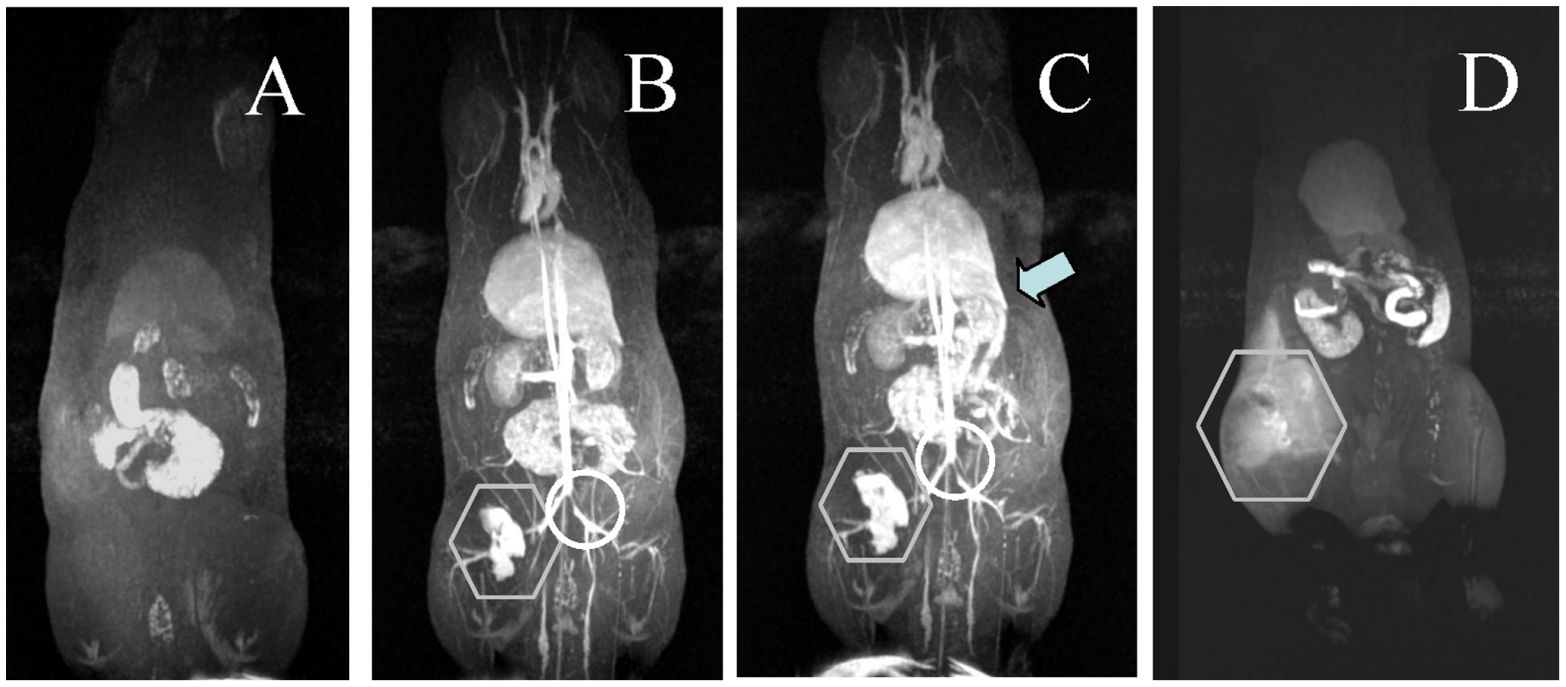
Time course of Gd-enhanced MR images for Gd-lipid nanoparticles. The rats were administered intravenously with 0.01 mmole/kg of Gd-lipid nanoparticles. The MR images at indicated time were collected with a 3T MR scanner. The animal was also scanned before dosing (panel A), 5 min (panel B), 15 min (panel C) or 24 hr (panelD) after Gd nanoparticle dosing. By 15 min (C), appearance of high positive contrast due to Gd elimination into the bile which connects to the intestine (arrows) and the gut is apparent. Also by 24 hrs, all the Gd-lipid nanoparticles were eliminated (panel D). Please note that the image for 24 hr did not line up due to different animal position. The contrast at 24 hr is found mainly in the GI tract, some of which are contributed by fatty materials as well. Also, due to leakage at the site of Gd administration 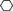 at the left femoral vein, Gd localization is apparent as a high contrast area. Please note the vaso-restriction ○ clearly apparent in panels B & C.

While Gd-LNP are not cleared by the kidney or accumulated in the bladder (Figure 2C–E), it is essential to define the route of Gd elimination. To do so, we first performed a time course MR image evaluation. The rat administered 0.01 mmole/kg Gd-LNP produced high resolution images within 5 minutes in all vasculature and highly perfused organs (Figure 3B). Gd-LNP appeared concentrated within blood vasculature, even within the kidney and liver (Figure 3C). By 15 minutes, Gd-LNP began to appear in the gut and could be traced to the bile duct in the liver. Furthermore, this process appeared complete within 24 hours, suggesting Gd-LNP that lack any cell or tissue binding ligands on their surface exhibited high intensity in blood without prolonged tissue exposure (Figure 3D). This image data suggests that Gd remains stably associated with DTPA-PE in lipid nanoparticles such that they are subject to biliary, instead of renal elimination. Should DTPA-PE in lipid nanoparticles metabolize to DTPA in liver or blood vasculature, we might have detected Gd or Gd-DTPA liberated from lipid nanoparticles as free form, appearing as positive contrast images in the kidney and bladder, which did not occur.

**Figure 4:**
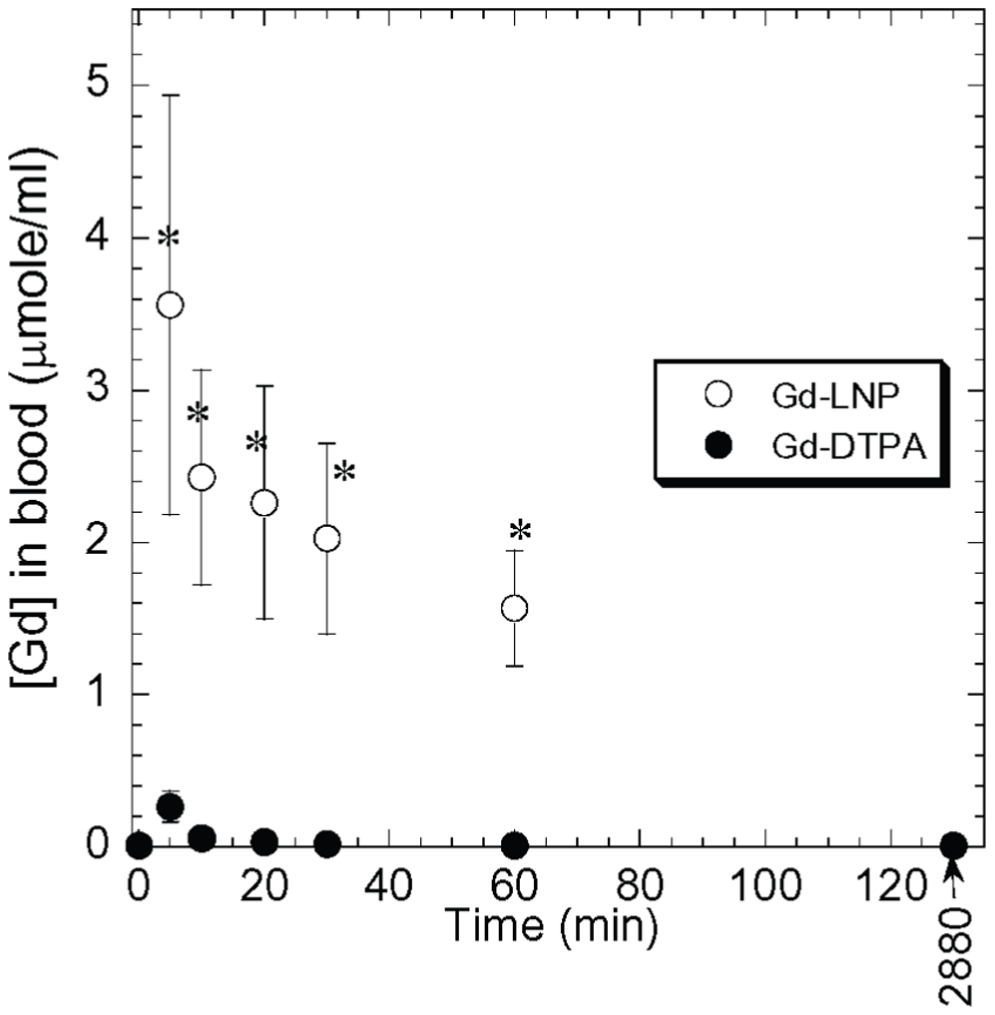
Time-course blood Gd kinectics in rats. Rats were intravenously given 0.5 mmole/kg of ^125^Gd either in Gd-lipid nanoparticles (Gd-LNP) or soluble Gd-DTPA and Gd concentrations in blood were analyzed and presented as µmole/ml. Data presented were mean ±a S.D of n = 4 per treatment group. * Indicates p<0.05 by student T test.

**Figure 5:**
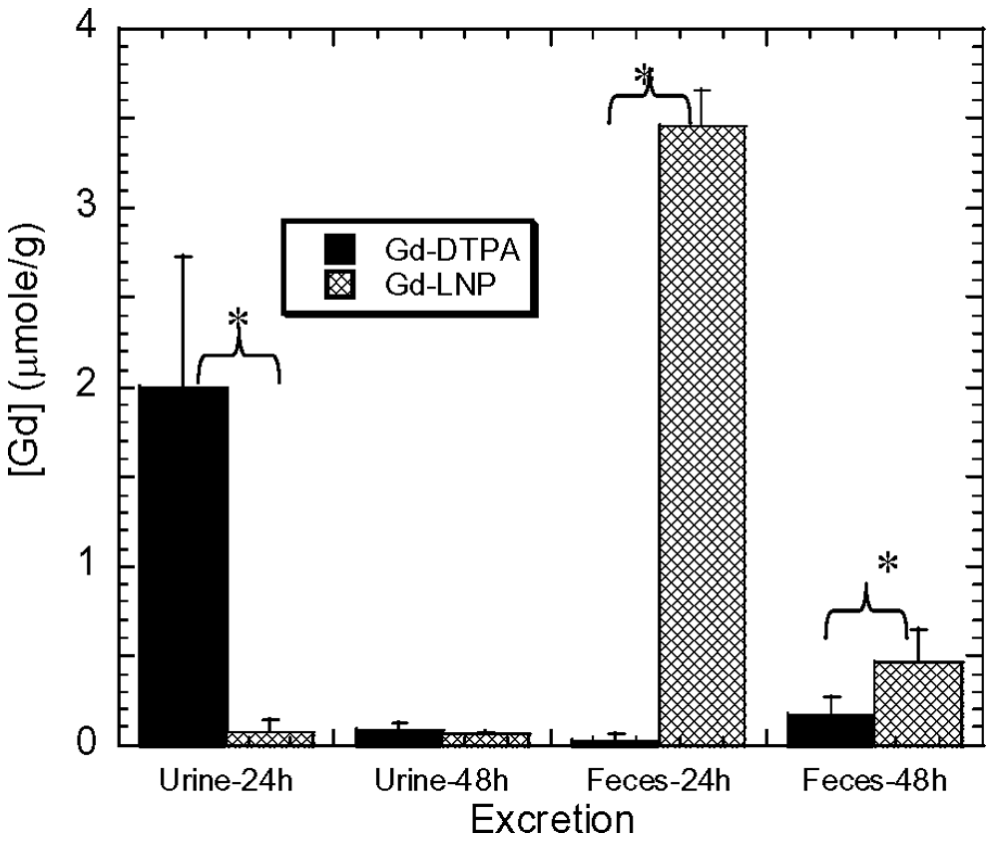
Comparison of Gd in LNP (hatched) vs soluble Gd-DTPA (filled) formulation excreted in feces and urine at 24 and 48 hrs. Rats were intravenously given 0.5 mmole/kg of ^125^Gd either in Gd-lipid nanoparticles (Gd-LNP) or soluble Gd-DTPA. The urine and feces collected from rats housed in metabolic cages were analyzed at 24 and 48 hr. Data were expressed as mean ±a S.D. in µmole/g [Gd] for each treatment group. * Indicates p<0.05 by student T test.

To further characterize the Gd clearance in Gd-LNP, we used ^153^Gd-labeled Gd-LNP in preliminary comparative pharmacokinetics and tissue distribution studies focusing on time-course blood kinetics, major organ distribution, excretion and residual Gd in muscle and skin tissues. As expected, rats (500 g; n = 4/group) administered with 0.05 mmole of Gd in LNP exhibited much higher blood Gd concentrations than with the Gd-DTPA formulation (Figure 4). The half-life of Gd-LNP and Gd-DTPA in the blood is estimated to be 45.7 and 6.1 mins. Analysis of urine and feces for Gd concentrations at 24 and 48 hrs revealed the distinct differences between soluble Gd-DTPA and Gd-LNP. Rats administered with Gd-DTPA almost exclusively eliminated Gd through the renal route and Gd appeared in urine; while those that received Gd-LNP eliminated Gd in feces through the biliary route (Figure 5). It is interesting to note that much higher concentrations of Gd excreted at both 24 and 48 hr post administration in feces (an end result of biliary excretion) in rats administered with Gd-LNP than those treated with Gd-DTPA (Figure 5), suggesting that Gd-LNP may be more effective in removing Gd from the body. These data confirmed the MRI data in Figure 3.

At 48 hrs post-exposure, residual Gd was found in the lung, liver and kidney for Gd-LNP; and for Gd-DTPA, most Gd was found in the bladder. Analysis of skin and muscle tissues revealed site-dependent highly variable skin and muscle accumulation in rats administered with Gd-DTPA, and much less variable and lower residual Gd concentrations were detected in rats administered with Gd-LNP. We also performed a preliminary behavioral toxicity study with mice and looked for skin agitation and lethargy with increasing doses of Gd in the LNP formulation. We used Magnevist and Omniscan for comparison. With as low as 0.02 mmole/kg Gd dose, mice began to exhibit skin agitation within 15 min for Omniscan and began to show signs of lethargy in 10–15 min. Gd given in Magnevist (Gd-DTPA) formulation exhibited a lower degree of skin agitation and mice were not lethargic with up to 0.2 mmole/kg dose. Under the same conditions and with up to 0.4–0.6 mmole/kg Gd-LNP, mice did not exhibit any signs of skin agitation. Collectively, these preliminary data suggest that Gd remains stably associated with LNP and thus no significant fraction of Gd is eliminated through the renal route (which is the main route of Gd-DTPA elimination). These data are also consistent with the MRI data presented in Figures 2–3.

## Discussion

Taking advantage of our ability to stably express Gd on the LNP surface via DTPA-PE chelate, we have identified a Gd-LNP formulation that exhibits about 33-fold higher *r_1_* relaxivity than the Gd-DTPA-BMA formulation. The high longitudinal *r_1_* relaxivity exhibited by these lipid nanoparticles is within experimental error of the maximum range predicted by simulation of Bloembergen and Morgan theory for paramagnetic systems [6], [7]. The review by Woods et al [6] estimates the maximum Gd *r_1_* based on computer simulation for Gd to be 110–120 mM^−1^*s^−1^. Single-wall carbon nanotubes (20–100 nm) loaded with Gd were reported to exhibit 160–174 mM^−1^*s^−1^ [8]. However, the shape and composition of carbon nanotubes may not be biocompatible.

The longitudinal relaxivity of Gd-LNP is also much higher than other lipid nanoparticles (diameter: 213–247 nm) described by Wickline and his colleagues (*r_1_* = 12.7 mM^−1^*s^−1^) [9]. Their *r_1_* values were much lower than observed with those coated with mPEG_2000_ (Table 1). While others have explored DTPA-PE in lipid vesicles or liposomes to enhance relaxivity of Gd-DTPA, reported *r_1_* values were around 10–12 mM^−1^*s^−1^ [4], [10]. For this Gd-lipid nanoparticle expressing mPEG_2000_-PE, the apparent high relaxivity reported is more than 2.5-fold higher than those achieved with Gd-DTPA-DPC (Vasovist) exposed to human serum albumin. Serum albumin levels may depend on renal disease state [11], [12] under the most favorable conditions [13]. Much higher relaxivity was achieved with Gd-LNP expressing mPEG_2000_-PE independent of serum albumin or other serum proteins.

**Figure 6:**
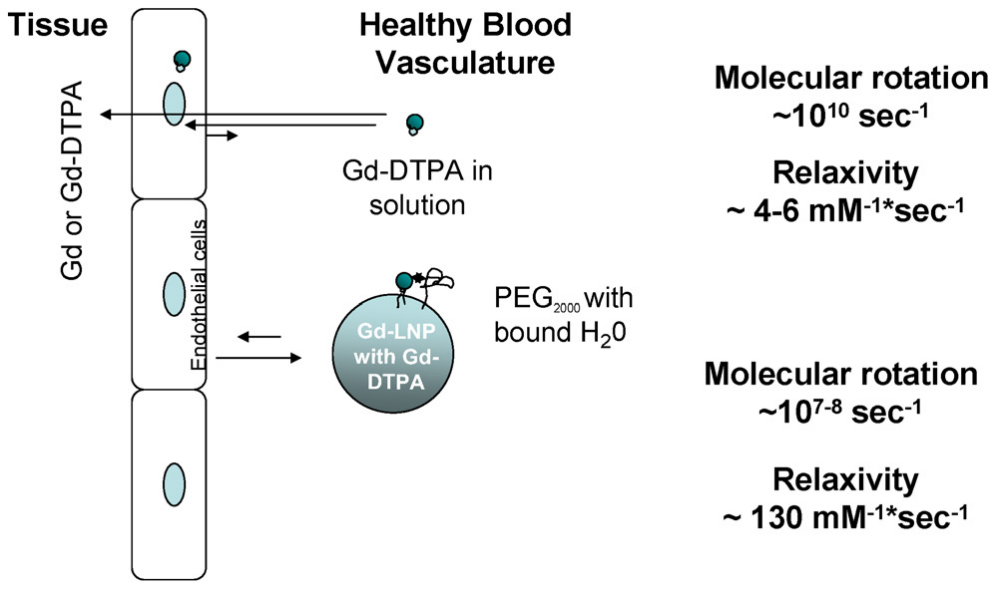
Schematic presentation of soluble and lipid nanoparticle associated Gd-DTPA molecular rotation and impact on relaxivity as well as their retention in healthy blood vasculature. The medium circular Gd and small circular DTPA chelate in solution or H_2_O exhibit high molecular rotation (∼10^10^ sec^−1^) that influences water proton relaxation, which typically produce 4–6 mM^−1^*sec^−1^ relaxivity. When Gd-DTPA is bound to lipid nanoparticle surface with surface bound water molecules attracted by PEG and Gd's molecular rotation is reduced to ∼10^7–8^ sec^−1^, relaxivity is greatly increased to about ∼130 mM^−1^*sec^−1^ (not drawn to scale). Gd in Gd-LNP also reduces the cellular uptake and thus lowers the cellular and tissue accumulation in non-pathogenic tissues and cells which may relate to clinical presentation of fibrosis.

While the exact mechanisms leading to the observed improvement in the longitudinal relaxivity remain elusive, it is possible that the surface bound water on lipid nanoparticles through mPEG_2000_ greatly reduces molecular rotation. As schematically presented in Figure 6, about 2–3 order of reduction in molecular rotation may translate to 22–33-fold enhancement in relaxivity. This hypothesis is consistent with the data presented in Table 1, where a change in surface hydration and varying PEG polymer size provided significantly different longitudinal relaxivity. Regardless, at the optimal PEG polymer size, mPEG_2000,_ Gd-LNP produced dramatically improved longitudinal relaxivity and also retained a majority of Gd in vasculature under normal conditions. A combination of reduced cellular and tissue uptake and enhanced longitudinal relaxivity likely contributed to the overall improvements in contrast potency (Figure 6). These and other mechanisms remain to be evaluated.

It is important to emphasize that Gd-contrast is almost completely eliminated by 24 hours, and that most of the Gd-LNP contrast was detected in the gut. In prior studies of liposomes expressing PEG, there is significant uptake and retention of PEG-liposomes in Kupffer cells in the liver and macrophages, and at least 20% of the injected dose remained in the liver at or beyond 50 hours in mice [3]. While detailed tissue localization studies are planned, the image data suggests that no significant Gd-LNP were detectable in the spleen or the liver by 24 hours (Figure 3). The distinct *in vivo* disposition profile between traditional liposome formulations and the Gd-LNP composition described here is also consistent with the unique morphology of Gd-LNP detected by electron microscopy (Figure 1B–C). While it needs to be validated, the current Gd-LNP with diameter ∼70 nm is far greater than the 50–70 kDa (d<3–4 nm) renal filtration threshold for globular proteins in humans. As retention of free or chelated Gd molecules in the kidney and tissues are proposed as one of the root causes of NSF in patients with renal diseases or impairment, the Gd-LNP hold promise both to improve effectiveness of contrast enhanced MRI as well as safety of Gd use in this patient population.

More importantly, a more complete clearance of these lipid-nanoparticles also suggests that background non-specific exposure or accumulation of these lipid-nanoparticle carriers in tissues is low. Thus, by attaching targeting molecules or peptides, these lipid nanoparticles could provide high specificity and low off-target accumulation for targeting to tumor receptors accessible through blood, i.e. leaky tumor-associated vasculature. Given the low degree of non-specific tissue accumulation, targeted lipid nanoparticles could be developed further as a better molecular imaging agent and drug loaded targeted nanoparticles could provide much higher therapeutic indices than classical liposomes or nanoparticles using other matrices.

The specific application of the Gd-lipid nanoparticles as a contrast agent remains to be directly demonstrated. However, it is likely that with a 45 min, instead of 5 min half-life, it could be used in a single dose instead of up to three injections to provide whole body MRI scans for detecting atherosclerosis. In addition, with the high resolution and low dose needed to detect vasculature in detail, it is likely to provide early detection of pathogenic conditions in highly perfused organs such as the lung, liver, kidney and microhemorrhage in the brain. Due to its movements, a different MR sequence may be needed for MR imaging of the lung. These and other potential applications are beyond the scope of this report and are under our current investigation.

In this report, we employed a linear chelate DTPA for proof-of-concept studies. The macrocyclic chelate DOTA can extend the DTPA, as a linear chelate, binding affinity of Gd about 3–4 orders of magnitude. The increased binding affinity of macrocyclic chelate to Gd has been proposed as one of the mechanisms to reduce clinical toxicity due to release of free Gd into cells and tissues. It is conceivable that we can further extend the DTPA in our Gd-LNP using macrocyclic chelating agents such as DOPA-PE, instead of DTPA-PE. These and other approaches to further stabilize the Gd association to the Gd-LNP are currently under our investigation. Regardless, the current formulation of Gd-LNP appeared to reduce non-specific tissue uptake and provided improvement in the extent of efficient elimination from the body.

In summary, our results indicate that an approximate 22–33-fold lower dose of current Gd contrast agents could be used to achieve similar or better whole-body image resolution for medical diagnosis. The enhancement in contrast potency achieved with Gd-LNP enable the use of only 3% or less of current clinical Gd dose needed for MRI procedures with potentially better vascular imaging capabilities. Furthermore, the rapid onset and unique clearance properties of Gd-LNP could also be adapted for system biology profiling of *in vivo* drug target distribution for new molecules that are identified as part of global/international efforts in pharmacogenomic and drug/target discovery research.

## Materials and Methods

### Materials

Phospholipids, 1,2-distearoyl-sn-glycero-3-phosphocholine (DSPC) and 1,2- distearoyl-sn-Glycero-3-phophoethanolamine-N-DTPA (DTPA-PE) were purchased from Avanti Polar Lipids (Alabaster, AL). 1,2-distearoyl-sn-glycero-3-phosphoethanolamine-N-[poly (ethylene glycol)_MW_] (mPEG-DSPE) was obtained from Genzyme Pharmaceuticals (Cambridge, MA). Gadolinium (III) chloride hexahydrate (Gd^3+^) and calcein were purchased from Sigma Chemical Co. (St. Louis, MO). Clinical Gd contrast agents were purchased from clinical pharmacy. Other ingredients were of analytical grade or higher.

### Preparation and characterization of Gd-lipid nanoparticles

The Gd-DTPA lipid nanoparticles were prepared by mixing DSPC:DTPAPE:mPEG-DSPE (9∶1∶1 mole ratio), were dissolved in chloroform, dried into a thin film under N_2_ and placed in a vacuum overnight. The same lipid composition without mPEG-PE were prepared as Gd-DTPA-liposomes. The phosphate buffered saline (PBS, pH 7.4) was added to the film and either sonicated or extruded through 50 nm polycarbonic filter at 60°C. To prepare Gd bound to lipid nanoparticles, the nanoparticles in suspension were mixed with Gd^3+^ (1∶1 DTPA-PE:Gd mole ratio) for 20 minutes. The same method was used to prepare ^153^Gd labeled Gd-lipid nanoparticles where Gd^3+^ was replaced with ^153^Gd^3+^. For comparison, water soluble commercial agents such as Gd-DTPA-BMA and Gd-DTPA were also included. The lipid particle diameter was determined with a Malvern Zetasizer 5000 photon correlation spectroscopy (Malvern Instruments, PA), and expressed at mean ±a SD.

### Magnetic Resonance Measurements

#### *In vitro* measurements

The relaxation time T_1_ was measured using the standard spin-echo sequence on a 3T MR scanner with a volume head coil as RF receiver operating at 37°C. For T_1_ measurements, TE (echo time) was fixed to 9 ms and seven TR (repetition time) were 133, 200, 300, 500, 750, 1000 and 2000 ms, respectively. For T_2_ measurements, TR was fixed to 2000 ms and four TE were 15, 30, 45, and 60 ms, respectively. The imaging intensities were fitted to obtain the corresponding T_1_ and T_2_ values for each concentration of Gd. These concentration dependent T_1_ values were plotted versus Gd^3+^ concentration from which the rising curve was fitted by linear regression to estimate apparent molar relaxivity constant r_1_ and r_2_. The covariant of r_1_ and r_2_ data were 15% or lower.

#### *In vivo* rat imaging studies

All animal experiments were performed with approved protocol # 2372-05 from the University of Washington Institutional Animal Care and Use Committee. Rats under anesthesia were implanted with an intravenous catheter in the femoral vein. They were given indicated doses of Gd-chelate or Gd-lipid nanoparticle preparations in 0.4 ml. MR images were collected sequentially at indicated time points with a 3T MR scanner (Acheiva, Philips Medical Systems, Best, Netherlands) using a small quadrature birdcage radiofrequency receiver coil. Axial, 2D T_1_-weighted, turbo spin-echo of the whole body was performed of each rat (TR/TE  = 750/6.4 ms, TSE factor 2, 500×470 µm in-plane resolution, 2-mm contiguous slice thickness). In addition, coronal 3D RF-spoiled, fat suppressed, gradient echo images were acquired (TR/TE/Flip  = 19/1.55 ms/40 degrees, in-plane voxel size  = 550×50 µm, 1.2 mm slice thickness). A control marker of Omniscan doped water was included in the imaging field of view to normalize signal intensity of each set of images across animals.

### Statistical Analysis

Data are presented as the mean ± SD. Statistical significance was evaluated either by unpaired Student's t-tests (two-sided) or one way ANOVA using SigmaPlot software (Systat, San Jose, CA).
